# Artificial antigen-presenting cells: the booster for the obtaining of functional adoptive cells

**DOI:** 10.1007/s00018-024-05412-y

**Published:** 2024-08-31

**Authors:** Jing Li, Weilin Zhou, Wei Wang

**Affiliations:** Department of Biotherapy, State Key Laboratory of Biotherapy and Cancer Center, West China Hospital, Sichuan University, and Collaborative Innovation Center for Biotherapy, Chengdu, 610041 People’s Republic of China

**Keywords:** Adoptive cell therapy (ACT), Cancer immunotherapy, Artificial antigen-presenting cells (aAPCs), Challenge and development

## Abstract

Adoptive cell therapy (ACT) achieves substantial efficacy in the treatment of hematological malignancies and solid tumours, while enormous endeavors have been made to reduce relapse and extend the remission duration after ACT. For the genetically engineered T cells, their functionality and long-term anti-tumour potential depend on the specificity of the T cell receptor (TCR) or chimeric antigen receptor (CAR). In addition, the therapeutic benefit is directly to sufficient activation and proliferation of engineered T cells. Artificial antigen-presenting cells (aAPCs), as powerful boosters for ACT, have been applied to provide sustained stimulation of the cognate antigen and facilitate the expansion of sufficient T cells for infusion. In this review, we summarize the aAPCs used to generate effector cells for ACT and underline the mechanism by which aAPCs enhance the functionality of the effector cells. The manuscript includes investigations ranging from basic research to clinical trials, which we hope will highlight the importance of aAPCs and provide guidance for novel strategies to improve the effectiveness of ACT.

## Introduction

Adoptive cell therapy (ACT) is a promising new treatment option for hematological diseases and solid tumours with limited treatment options [1–3]. ACT involves inducing, activating, or genetically engineering anti-tumour lymphocytes from patients or healthy donors in vitro, then returning them to the patients to recognize and eliminate tumour cells. Generating sufficient functional engineered T cells is a prerequisite for achieving promising therapeutic efficacy, which is largely determined by the in vitro activation and expansion steps. In vivo, these processes are enhanced by professional antigen-presenting cells (APCs), such as dendritic cells (DCs), macrophages, and B cells [4–7]. However, the isolation and purification of autologous APCs can be labour-intensive. Furthermore, their utilization is often hindered by the lack of consistency and standardization in terms of cell number and phenotype. In this context, artificial antigen-presenting cells (aAPCs) have been developed with the aim of enabling more efficient and reproducible ex vivo activation and expansion of T cells. aAPC refers to both acellular and cell-based aAPC [8–10]. In this manuscript, we compare the advantages and limitations of cell-based and acellular aAPC. We argue that cell lines, once generated and stored, offer an advantage over autologous APC, which cannot be stored for long periods of time, by providing a long-term, reliable source of reagents for T cell generation or expansion. One significant advantage of cellular aAPCs over non-cellular is their capacity to effectively emulate the natural physiologic interaction between T cells and APCs. These interactions are essential for aAPC signalling of T cells [11]. We believe that cell-based aAPC, offering considerable promise, is desirable for immunotherapy. To date, a variety of aAPCs with different designs and properties have been synthesized and demonstrated to be highly efficacious in T cell activation for clinical applications. Despite considerable progress, the current development of aAPCs still faces challenges, including limited specificity and effectiveness, overstimulation, rapid exhaustion, and a lack of sustained cytokine release. To enhance the efficacy and accessibility of ACT, the development of a more efficient and safer APC represents a pivotal and immediate objective. Herein, we summarize recent advances in cell-based aAPC for both ex vivo and in vivo applications in ACT.

## Natural biological signals of the immune system utilized by aAPC

**Fig. 1:**
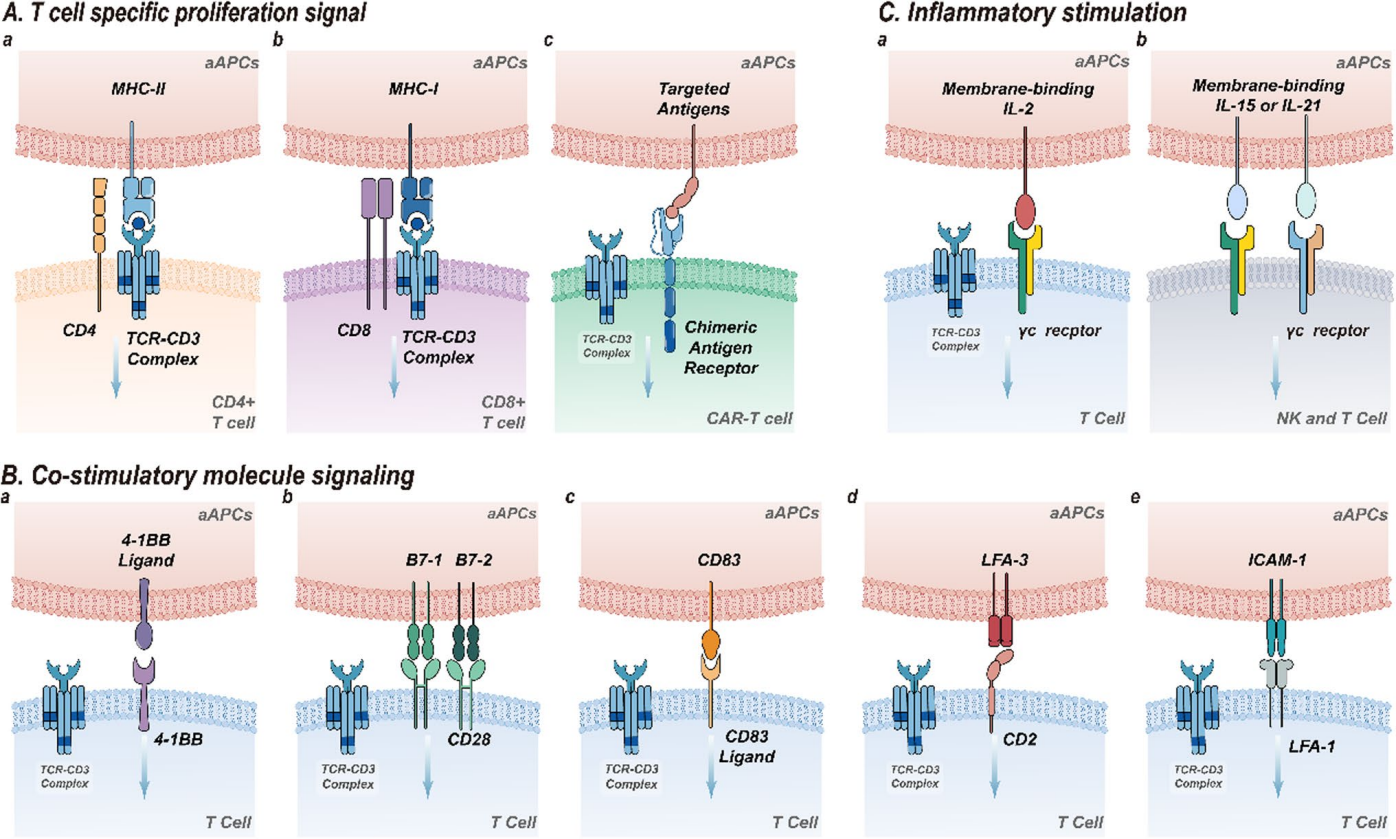
Signals on aAPC to meet the therapeutic needs of adoptive T cells. **A** MHC molecules and target surface antigens provide specific proliferative signals for T cells. **B** Co-stimulatory molecules are an essential signal for T cell proliferation and differentiation. **C** Different exogenous cytokines and membrane-bound cytokines promote the proliferation and differentiation of immune cells with different functions. *MHC* major histocompatibility complex

The activation of T lymphocytes is a hallmark of the immune response [12]. Dual-signal stimulation is required to trigger T cell activation, proliferation and differentiation into effector T cells. The initial signal is mediated by the major histocompatibility complex (MHC) peptide complex (signal 1) and amplified by adhesion molecules [13]. The co-stimulatory signal (signal 2) is generated by the interaction between APCs and T cells [14, 15], which can enhance the T cell receptor (TCR) signal. The surface synergistic stimulatory molecules of APCs play an essential role in this process. Cytokines (signal 3) are also pivotal biological determinants of T cell expansion, survival, and function [16, 17]. The aforementioned T cell activation signals can be reconstituted by expressing the appropriate ligand combinations in the aAPC (Fig. 1). Subsequently, we will present how distinct combinations of ligand constructs on aAPC can regulate the functions of T cells.

### Signal 1: T-cell specific proliferation signal

The function of the MHC or human leukocyte antigen (HLA) is to bind and present antigen peptides for recognition by T cells. MHC class I molecules are responsible for the combination and presentation of endogenous antigen peptides following proteasome treatment in the endoplasmic reticulum. The peptide is combined with MHC class I molecules to form a complex (pMHC), then presented to the cell surface [18]. After the TCR recognizes a specific pMHC, CD8^+^ T cells are activated and subsequently transformed into active cytotoxic T lymphocytes. These cells are responsible for the killing of target cells. MHC class II molecules are responsible for the presentation of exogenous antigen peptides to CD4^+^ T cells, allowing CD4^+^ T cells to activate, proliferate and express appropriate lymphokines [19]. Multiple studies have demonstrated that aAPCs expressing HLA-class I molecule can maintain the proliferation of antigen-specific CD8^+^ cytotoxic T lymphocytes (CTLs). Furthermore, these CTLs are able to identify and effectively kill tumour cell targets when HLA-restriction is in place [20]. This strategy has been validated in the clinical trial stage. The MART1-specific CTL generated by aAPCs, transduced with CD80, CD83 and HLA-A * 0201 can persist in vivo and induce anti-tumour response [21, 22]. Several pieces of evidence show that CD4^+^ T cells contribute to the generation of optimal anti-tumour CD8^+^ T cell responses and the establishment of long-lasting memory [19, 23]. Consequently, researchers have developed aAPC expressing MHC class II molecules for the expansion of CD4^+^ T cells [24, 25]. For instance, aAPCs engineered based on K562 cells express different single HLA-DR alleles, invariant chains, and HLA-DM. These aAPCs can absorb soluble proteins by endocytosis and process and present peptides [26]. The novel tool was employed to amplify long-acting CD4^+^ T cells with specificity for multiple antigens, while preventing the growth of Foxp3^+^ regulatory T cells [26]. Chimeric antigen receptor T (CAR-T) cells harness extracellular single-chain variable regions (scFv) to recognize tumour antigens, initiate intracellular signals and eliminate tumour cells. The scFv is composed of the variable heavy chain and variable light chain from the specific antibody. It is capable of recognizing tumour cell surface antigens, leading to MHC-independent activation of T-cells. The genes cargo encoding tumour antigens were introduced into aAPC, which facilitates the proliferation of CAR-specific T cells. For example, multiple aAPCs expressing CD19 molecules have been shown to expand CD19-specific CAR-T cells [27–32]. Compared to the method of T cell expansion with γ-irradiated feeder cells, expansion with CD19-expressing aAPCs was able to proliferate T cells with increased CAR expression and increased cytolytic activity [27].

### Signal 2: co-stimulatory molecule signal

Engagement between TCR and p-MHC complexes is necessary, but not sufficient, for the activation of T cells [33, 34]. In fact, triggering the TCR alone usually leads to limited expansion, followed by unresponsiveness after reencountering antigen. To effectively activate T cells, co-stimulatory molecule signals have to be provided to T cells. Co-stimulatory signaling plays a critical role in modulating T-cell activation, differentiation, effector function, and survival. Thus, numerous co-stimulatory molecules (such as CD28, 4-1BB, CD83 and adhesion molecules) can be applied to the aAPC for enhancing the immunotherapy. The next section reviews the biological functions of different co-stimulatory signals and the effects of co-stimulatory molecular signals expressed on aAPCs for adoptive T cells.

#### Co-stimulatory signals mediated by CD28

Multiple biological functions, including T cell expansion, IL-2 secretion, and Th1 differentiation, were mediated by the interaction between CD28 and corresponding receptor [35]. Therefore, most aAPCs express the related receptor CD80(B7-1) or CD86(B7-2) of CD28 [20, 36–38]. Data suggest that aAPCs expressing CD80 were crucial to sustain the proliferation and re-stimulation of CD19-specific CAR-T cells [39]. Intercellular cell adhesion molecule-1 (ICAM-1) provides synergistic signals via LFA-1 molecules expressed on T cells. Preclinical evidence indicates that ICAM-1 expression results in weak antigen-specific CTL stimulation and limited tumour protection [40]. In addition, ICAM-1 has extensively documented its synergistic effect with CD28. A study co-transfected B7-1 and ICAM-1 into *Drosophila* cells to produce the novel aAPCs that trigger sustained T cell proliferation, cytokine secretion and specific cytotoxicity [41]. A research team modified a mouse fibroblasts-based aAPC that expressed an HLA-peptide complex along with B7-1, ICAM-1, and LFA-3. The strategy elicited strong stimulation and consistent proliferation of CTLs [42].

#### Co-stimulatory signals mediated by 4-1BB

4-1BB is an inducible receptor derived from the tumour necrosis factor (TNF) receptor superfamily [43]. The ligand named 4-1BBL, expressed on a fraction of DCs constitutively, is inducible in macrophages, B cells, and T cells. In the presence of TCR engagement, 4-1BBL or agonist monoclonal antibodies signal through 4-1BB to trigger T cell expansion, produce cytokines, and prevent activation-induced cell death (AICD) [44, 45] To determine which co-stimulatory molecules could promote the expansion of CD8^+^ T cells with effective functions, a series of different aAPCs were designed to improve adoptive T cells [38]. In contrast to aAPC expressing CD80 alone, aAPC expressing 4-1BBL alone can maintain a longer proliferation of CD8^+^ T cells. In addition, another research demonstrated that the addition of 4-1BBL can maintain the TCR diversity of T cells and reduce apoptosis of CD8^+^ T cells [46]. It is worth noting that the effect of aAPCs co-expressing CD80 and 4-1BBL is better than that of APCs expressing only one agonist molecule [38]. The 4-1BB signal is essential for the amplification of CD8^+^ T cells, but the long-term growth of CD4^+^ T cells does not depend on the 4-1BBL signal [47]. Of note, compared to aAPC expressing 4-1BBL or B7-1 alone, aAPC co-expressing 4-1BBL and B7-1 enhanced the effector CTL anti-tumour response [48].

#### Co-stimulatory signals mediated by CD83

Human CD83 is the signature of mature DCs, and also expresses on activated B cells and T cells [49]. The downregulation of CD83 in DCs results in a reduction in their capacity to induce T cell proliferation. Furthermore, DCs downregulating CD83 induce a reduction in interferon (IFN-γ) secretion by activated T cells. CD83 ligands have been shown to induce the long-term expansion of CD8^+^ T cells, with a preferential enrichment of antigen specificity [20]. Furthermore, CD83 has been demonstrated to be crucial for the longevity of B cells and CD4^+^ T cells [50]. The precise impact of CD83 on aAPC or T cells remains to be elucidated, and some studies indicate that CD83 may act in a synergistic manner with CD28 and/or 4-1BB signal. In general, CD83 is responsible for the preferential enrichment and long-term amplification of antigen-specific CTLs, while this process also requires the participation of CD80 [20, 22]. The co-expression of aAPC with CD80 and CD83 also inhibited cell apoptosis in CTL compared to aAPC expressing CD80 only.

#### Adhesion molecules

The stable formation of immune synapses (IS) is one of the key factors in T cell activation. Adhesion molecules play a pivotal role in establishing a stable IS. As a crucial adhesion molecule, the ICAM-1 is also frequently used in the aAPC system. The importance of ICAM-1 and its synergy with CD28 has been widely demonstrated [41, 42]. Some studies suggest that the expression of additional co-stimulatory and/or adhesion molecules on aAPC may further enhance the capacity to promote antigen-specific T cell population proliferation. These adhesion molecules are highly expressed on DCs and can induce naive T lymphocytes. The major roles of B7-1, ICAM-1, and LFA-3 in co-stimulating CTLs have been documented [51, 52]. *Drosophila* cells transducing ICAM-1 and B7-1 transform into highly effective aAPCs, inducing robust proliferative and cytokine production. [41]

Overall, CD28 co-stimulatory signals were able to induce sustained T cell proliferation, cytokine secretion, and specific cytotoxicity. The 4-1BB co-stimulatory signaling is important for sustained T cell proliferation. In addition, CD83 co-stimulatory signals act in concert with other co-stimulatory molecules to inhibit the exhaustion of T cells. Adhesion molecules can also synergize with other co-stimulatory signals to promote the formation of IS and further promote T cell proliferation and cytokine secretion. The mechanism of synergistic interaction between co-stimulatory molecules remains unclear. At present, it has not been demonstrated whether the interaction of co-stimulatory molecules activates two discrete signaling pathways or initiates a unique signal transduction pathway. Further investigations are needed to demonstrate the importance of the expression of different co-stimulatory ligands in aAPC for the induction of different functions in T cells.

### Signal 3: aAPCs armed with diverse cytokines

Cytokines are essential for promoting T-cell proliferation, survival, and anti-tumour function [16, 53]. IL-2 is a key cytokine regulating the innate and adoptive immune system. IL-2 has the ability to mediate and regulate multiple biological events, particularly in T cell proliferation, differentiation, and development [54]. IL-2 can stimulate natural killer (NK) cell proliferation and amplify cytolytic activity [55]. Antibody production and proliferation of B cells can also be induced by IL-2 [56]. However, IL-2 exposure can cause AICD [57], and the production of immunosuppressive Treg cells. Given the important role of IL-2 for immune cells, the vast majority of current protocols utilizing K562-aAPC as a T cell stimulator require the incorporation of IL-2 into the culture.

In addition to IL-2, another two γc family cytokines (IL-15 and IL-21), also play important roles in T cell growth and development. IL-15 has a significant effect on memory cells’ proliferation and anti-apoptosis [58]. IL-21 can stimulate cell proliferation, regulate gene expression and the differentiation of Th1 or Th2 cell [59, 60]. Both IL-15 and IL-21 are involved in maintaining and expanding memory CD8^+^ T cells, NK cells, NKT cells [61]. In the presence of exogenous IL-2, NIH-3T3 aAPCs have difficulty maintaining sustained T cell expansion [39]. The sustained proliferation of tumour-specific T cells can be maintained in the presence of IL-15, allowing the preparation of T cells on a clinical-use scale. The therapeutic potential of T cells expanded in the presence of IL-2 or IL-15 was also compared. The study has reported that T cells expanded in the presence of IL-15 had greater anti-tumour activity and eradicated established tumours in animal models. Recently, we noted that aAPC expressing tumour antigens can increase CAR-T cell-specific proliferation; however, persistent tumour antigen stimulation may result in faster CAR-T exhaustion [62]. The presence of exogenous IL-2, IL-9, and IL-21 alleviated the dysfunction of CAR-T cells and further promoted anti-solid tumour capacity of CAR-T cells.

It is also feasible to genetically design aAPCs to produce or express cytokines in a membrane-bound form. Clone#4 (CD19, CD64, CD86, 4-1BBL, and membrane junctions co-expressed on K562)was created to support the specific amplification of CD19-specific CAR-T cells. However, it also requires the presence of the external cytokines IL-2 and IL-21 [32]. aAPCs with membrane-bound cytokines are largely employed to stimulate NK cell proliferation. Common γc-chain cytokines such as IL-2, IL-15, and IL-21 play a significant role in the activation, maturation, and proliferation of NK cells. aAPCs expressing mbIL-21, unlike mbIL-15, supported the proliferation of NK cells and showed no evidence of senescence after up to 6 weeks of incubation [63–65]. The phenotypic and cytotoxicity of mbIL-21-amplified NK cells were comparable to those of mbIL-15-amplified cells. NK cells amplified with mbIL-21 have shown considerable cytotoxicity against all tumour cell lines. Additionally, NK cells after culture increased antibody-dependent cell-mediated cytotoxicity (ADCC), while maintaining the responsiveness to inhibitory killer cell immunoglobulin-like receptors (KIRs).

The impact of a single cytokine on adoptive cells has been extensively researched, yet the influence of distinct combinations of cytokines on the phenotype of adoptive cells remains uncertain. Furthermore, cell-based APCs can be employed as an efficacious instrument to investigate the impact of disparate cytokines on adoptive cells.

One of the key processes in achieving clinical efficacy of tumour-specific T-cell immunotherapy is that the APC presented signals from tumour cells to T cells, which activates tumour-specific T cells and enables them to lyse cancer cells. Understanding how different signals affect T cell fate helps researchers apply these signals to the preparation of aAPC, with a view to developing adoptive T cells with a more powerful anti-tumour function.

## Advantages and limitations of different aAPCs

**Fig. 2:**
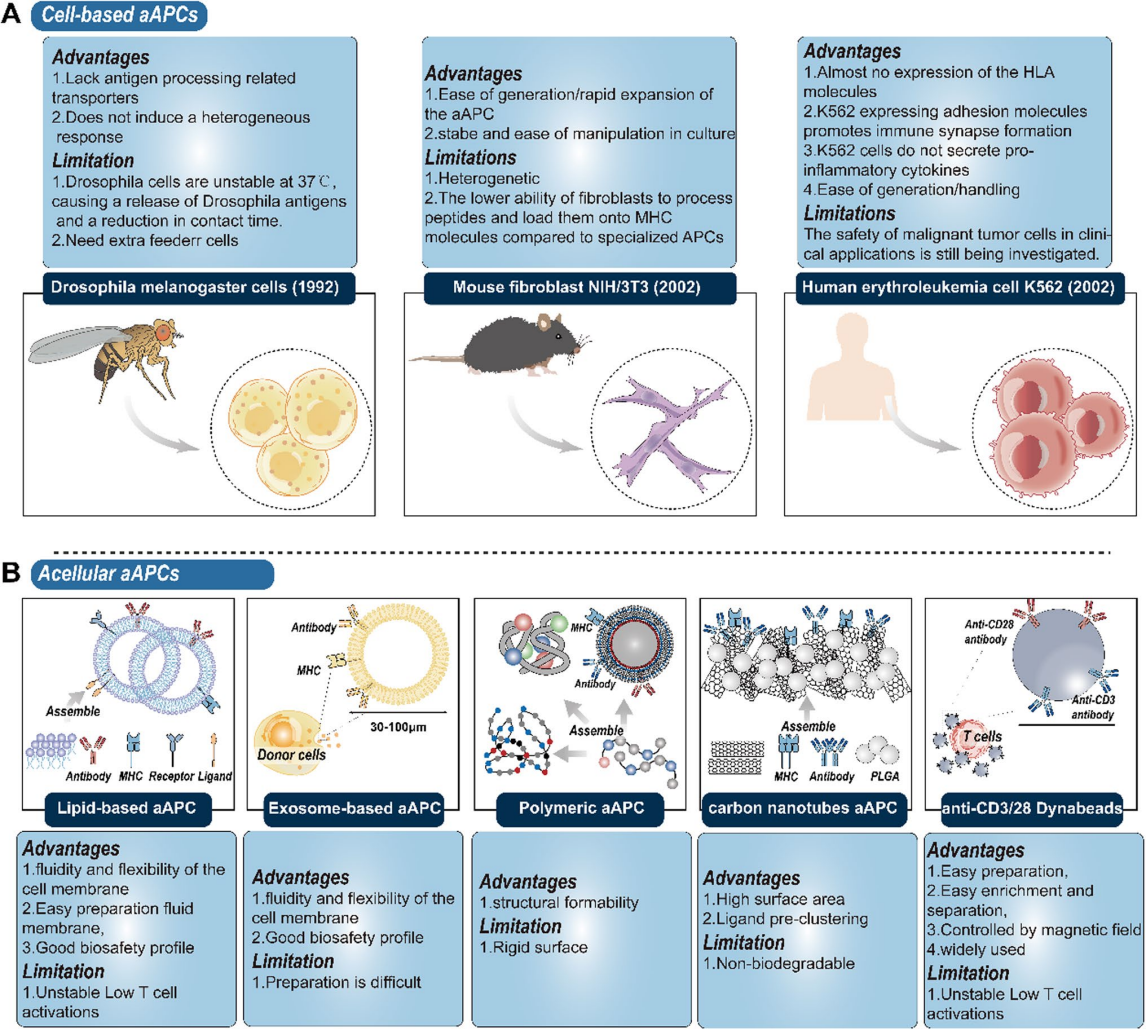
Classification, advantages and limitations of aAPC. aAPC is mainly divided into cell-based aAPC and non-cellular aAPC. **A** According to its different cytoskeleton, cell-based aAPC is classified into: *Drosophila* *melanogaster* cell, mouse fibroblast NIH/3T3 and human erythroleukemia cell K562-based aAPC. **B** Different non-cellular aAPCs, and their advantages and limitations

Several studies have demonstrated that the utilization of autologous APCs in ACT contributes to favorable clinical outcomes and minor adverse side effects [66, 67]. Those results highlight the promise of APCs in adoptive immunotherapy. Unfortunately, the applications of natural APCs over the years have also encountered some significant limitations (Table 1). The isolation and stimulation of autologous APCs in vitro is a time-consuming and costly process [68, 69]. Moreover, the quality of autologous APCs generated in vitro is uneven. Consequently, investigators have generated various types of aAPCs to better expand T cells. We review these aAPCs directly below. Artificial antigen-presenting systems encompass both cell-based (Fig. 2A) and acellular systems (Fig. 2B). aAPC prepared using distinct material compositions exhibits distinctive advantages and limitations. The following section will present the distinguishing characteristics of the various aAPCs. We contend that this can serve as a guide for researchers in applying aAPC in ACT.

### Cell-based aAPC

Specific cells must satisfy several requirements to be candidates (backbone cells), which will be used in the construction of the universal aAPCs. First, the induction of allospecific T cells by backbone cells should be absent or minimal if any does occur. Therefore, the backbone cells do not express any HLA class I or II molecules. Second, the candidate cells must be readily amenable to genetic manipulation and the expression of the manipulated genes must be stable. Third, it would be preferable for the backbone cell line to have a documented history of safe use in humans.

#### Drosophila melanogaster cells

One of the earliest cell-based aAPC systems employed *Drosophila* cells. Because *Drosophila melanogaster* cells do not possess antigen-processing-related transporters, they cannot present endogenous peptides to HLA class I molecules. *Drosophila* cells are unable to process endogenous antigens and do not induce allogeneic responses. Consequently, *Drosophila* *melanogaster* cells are considered to offer an outstanding exogenous peptide epitope [41, 70, 71]. *Drosophila*-derived aAPCs were transduced with HLA-A*0201 and pulsed with tyrosinase peptides to expand tyrosinase-specific CD8^+^ T cells, which were infused into melanoma patients subsequently [72]. Washing the CTL product prior to refusion effectively eliminated aAPC, and no adverse effects associated with insect cells were observed in any of the patients. However, this limits their applicability in studies requiring antigen processing. The application of a *Drosophila* *melanogaster* cell-derived aAPC presents a significant flaw. The aAPC of *Drosophila melanogaster* cannot survive at 37 ℃ without the aid of peripheral blood monoculear cell (PBMC) feeder cells. [71] Scientists have therefore investigated other cells as alternative aAPC scaffolds.

#### Mouse fibroblast NIH/3T3

In contrast to *Drosophila* aAPC, NIH/3T3 cells exhibit the capacity for endogenous antigen processing. This has been observed to stimulate the CD8^+^ T cells to respond to exogenous antigens that have been loaded and transfected [73]. NIH/3T3 cells are commonly employed for the activation of mouse and human CTL because of multiple characteristics. The properties include facile expansion, sustained gene expression and antigen processing [40, 42, 74]. Mouse fibroblasts have the extra advantage of being more stable and easier to manipulate in culture than *Drosophila* cells. Furthermore, the reduced ability of fibroblasts to digest peptides and load them onto MHC molecules in comparison to professional APCs may promote the expression of certain HLA-polypeptide complexes by lowering the simultaneous expression of unrelated peptides [75, 76]. To illustrate, NIH/3T3 cells transduced to express CD80, 4-1BBL, and the prostate-specific membrane antigen (PSMA) were shown to effectively expand PSMA^+^ CAR-T cells [77]. The resultant T cells were observed to be effective in the treatment of PSMA-positive tumours in a humanized mouse model.

#### Human erythroleukemia cell K562

K562 serves as the scaffold for the cell-based aAPC due to several reasons [22]. First, K562 cells lack HLA expression, which prevents allogenic responses. At the same time, HLA-deficient K562 cells do not activate KIRs. Therefore, K562-derived aAPC are ideal for expanding NK cells [78]. Second, the cells also contain adhesion molecules that enhance T-cell–aAPC interactions. The fact that K562 cells do not secrete pro-inflammatory cytokines such as IL-12 or type I IFN may facilitate selective expansion of central memory T cells. Importantly, previous studies have demonstrated that K562 can stably express a wide range of molecules on the surface, suggesting that K562 is suitable for gene transduction [20, 28, 38]. As a consequence, K562-based aAPCs are currently the most widely utilized in cell therapies. K562 aAPCs genetically modified to express CD64, CD86 and CD137L were demonstrated to facilitate the expansion of NKG2D CAR-T cells from small volumes of peripheral blood samples [37]. CAR-T cells expanded with these aAPCs are capable of meeting the requirement for multiple transfusions in the treatment of solid tumours [37]. Stimulation of CD8^+^ T cells with peptide-pulsed aAPC produces a considerable number of functional CTLs [22]. These CTLs are capable of recognizing a wide range of tumour antigen. And CTLs expanding with aAPC are capable of lysing a variety of melanoma tumour lines that naturally express target melanoma antigens.

### Acellular synthetic aAPCs

#### Lipid-based aAPC and exosome-based aAPC

The fluidity and flexibility of the cell membrane facilitate the formation of immune synapses between APCs and T cells, thereby promoting T cell activation. In order to emulate the natural interactions between APCs and T cells, lipid-based aAPCs have been developed [11]. The preparation of lipid-based aAPC is a relatively convenient process, and it has been extensively employed for the expansion of clonal T cells and antigen-specific T lymphocytes [79, 80]. Nevertheless, the most notable drawback associated with lipid-based aAPC is instability, which may result in inadequate activation of T cells [81]. In order to improve the stability of lipid-based aAPC, lipid bilayer aAPC with solid particles as scaffolds, also known as a supported lipid bilayer, was developed [82, 83]. For example, aAPC prepared by encapsulating natural cell membranes on silica microbeads and latex microbeads can effectively stimulate T cell proliferation [84]. Exosomes are extracellular vesicles that can be produced by tumour cells or other types of cells [85–87]. They have been demonstrated to stimulate immune responses in vivo. Tumour cell-derived exosomes also necessitate the involvement of cells (such as DCs) for antigen presentation [85], whereas DC-derived exosomes do not. Exosome-like vesicles secreted by the *Drosophila melanogaster* aAPCs have been demonstrated to stimulate the IL-2 and IFN-γ production of mouse CD8^+^ T cells in vitro [88]. Exosomes retain the advantages of cell membrane scaffolding as cellular nucleus-less structures. Nevertheless, the potential of exosomes to support ex vivo T-cell expansion remains to be elucidated.

#### Polymeric aAPC

Polymers such as polystyrene beads and lactic-co-glycolic acid (PLGA) have also been employed in the generation of aAPCs [9, 89, 90]. Polymeric aAPCs not only immobilize molecules such as pMHC, CD3, CD28 on the surface, but also slowly release soluble cytokines [91]. Compared to the traditional method of adding exogenous cytokines, the slow release strategy has been proven to promote T cell proliferation more effectively [91]. Three signals essential for activating T cells are integrated into a single polymeric aAPC, which facilitates the rapid expansion of clonal T cells. The structural malleability of polymeric aAPCs is another advantage. By replacing round aAPCs with oval ones, the contact area between aAPCs and T cells can be increased [92]. The oval-shaped PLGA aAPC has been demonstrated to promote stronger T-cell proliferation in vitro and to stimulate stronger immune response in mice [93]. Nevertheless, a significant limitation of polymeric aAPC is its surface rigidity.

#### Inorganic aAPC

Inorganic aAPC Inorganic aAPC involves carbon nanotubes [94] and magnetic particles [95]. Magnetic beads were developed by Levine et al. to maximize the expansion of T cells in vitro [15]. Anti-CD3/CD28 dynabeads are the most widely used aAPCs for ACT. The product has been approved by the US Food and Drug Administration (FDA) for the activation and expansion of T cells [95, 96]. Carbon nanotubes are nanomaterials with a large surface area and can locally adsorb high concentrations of stimulatory ligands [97]. When anti-CD3/CD28 antibodies are adsorbed on carbon nanotubes, the antibodies aggregate and form antibody clusters in the concave region on the surface to promote T-cell activation [98]. However their application in vivo T-cell activation has been limited due to their cytotoxicity and poor biocompatibility [99]. It is necessary to remove the aAPC completely prior to infusion of T-cells, which presents a significant challenge for the quality control of adoptive T-cells.

**Table 1:** Advantages and limitations of different aAPCs

Types of aAPC	Advantages	Limitations
Acellular aAPC		
Lipid-based aAPC	Fluidity and flexibility of the cell membrane Easy preparation fluid membrane, good biosafety profile	Unstable Low T cell activation
Exosome-based aAPC	Fluidity and flexibility of the cell membrane, good biosafety profile	Preparation is difficult
Polymeric aAPC	Structural formability	Rigid surface
Inorganic aAPC	Carbon nanotubes: high surface area, ligand pre-clustering Dynabeads: Easy preparation, easy enrichment and separation, controlled by magnetic field, widely used	Non-biodegradable
Cell-based aAPC		
K562 cell-based aAPC	Almost no expression of the HLA molecules, expressing adhesion molecules promotes immune synapse formation, ease of generation/handling	Risk of tumourigenesis
NIH/3T3 cell-based aAPC	Ease of generation/rapid expansion of the aAPC, stable and ease of manipulation in culture	Heterogenetic, the lower ability of fibroblasts to process peptides and load them onto MHC molecules compared to specialized APC
*Drosophila* *melanogaster* cells-based aAPC	Does not induce a heterogeneous response Lack antigen processing related transporters	Self-elimination at 37 °C, peptide pulsing required Need extra feeder cell
Autologous APC	Minor adverse side effects	Time-consuming and expensive Equality of ex vivo-generated autologous APC can be variable

## Cell-based aAPC system in ACT

The complete cellular structure and the adhesion molecules expression of K562-based aAPC were able to promote the formation of the immune synapse. These properties were more effective in mimicking the physiological interaction between T cells and APC [22]. The interactions are critical for aAPC to signal to T cells [12]. The applications of acellular aAPCs have recently been extensively reviewed [81, 100, 101], and this review will focus on the use of cell-based aAPCs in ACT.

### Application of cell-based aAPC in CAR-T cell therapy

The preclinical or clinical amplification of CAR-T cells was mainly stimulated with microbeads coated with anti-CD3/CD28 antibodies. One limitation of this amplification method is that the expansion of T cells is not CAR-specific. The researchers compared anti-CD3/CD28 monoclonal antibody-coated beads with aAPC expressing mOKT3, CD80, and CD83 molecules [102]. The study discovered that brief stimulation of cell-based aAPCs, rather than long-term beaded stimulation, resulted in greater CD8^+^ T cell expansion. Transiently stimulated CD8^+^ T cells maintain the stem-like memory phenotype and secrete multiple cytokines more efficiently than long-stimulated T cells. Therefore, several groups have used aAPCs expressing CAR-target antigens to proliferate CAR-specific T cells [27–29, 37]. According to a research, aAPC increased the cytolytic potential of CAR-T cells by encouraging the selective proliferation of CAR^+^ T cells, even if the expansion rate was somewhat slower than that of CAR-T cells expanded using allogeneic feeder cells [27]. CD19^+^ 4-1BBL^+^ MICA^+^-K562 amplified CAR-T cells had not only strong selective amplification but also higher cytolytic activity [28]. Some studies showed that when stimulated by the supernatants of T cells activated by IFN- γ, TNF- α, CD3/CD28, K562 may upregulate the expression of MHC class I molecules [103, 104]. MHC class I negative K562 cells were generated by destroying the B2M motif in K562 cells using CRISPR/Cas9 technology [36]. These aAPCs could attenuate allogeneic immune responses while promoting robust antigen-dependent and CD19-specific CAR-T cell proliferation. A research team is generating clinical-grade CAR^+^ T cells with K562-based aAPC (clone#4). Clone#4-expanded CAR-T cells can be used to treat B-lineage lymphoid malignancies and B cell chronic lymphocytic leukemia [32, 105]. Monocyte contamination of the starting T cell source suppresses microbead-based CAR-T amplification, and in some cases causes production failure [106, 107]. To address this issue, aAPC was prepared from K562 low-density lipoprotein receptor (LDLR) knockdown cells for the activation and expansion of T cells [108]. The LDLR-knockdown aAPC amplified CAR-T cells more effectively than magnetic beads or inactivated PBMC. The survival and anti-tumour efficacy did not differ significantly between this aAPC and magnetic bead-expanded CD19^+^ CAR-T cells in vivo. The NKG2D ligand is expressed endogenously on K562 and increases upon irradiation. Tay et al. demonstrated how to generate clinically relevant NKG2D CAR-T cells by aAPC cells co-expressing CD64, CD86, and 4-1BBL [37]. This aAPC amplification produced the final cell therapeutic product with 89.2% ±10.2% NKG2D CAR^+^ T cells. The expression of exhaustion markers TIM-3 and LAG-3 on T cells decreased with culture time, but did not alter antitumour efficacy of CAR-T cells. Heparin II binding domain (HBD) is significant in helper gene transduction [109, 110]. In order to promote CAR gene transduction, K562 cells were co-transduced HBD, anti-CD3, and anti-CD28 scFv and 4-1BBL [111]. This aAPC supported efficient gene transduction and CAR-T cell expansion, and fewer CAR-T cells were exhausted than beads-amplified ones.

The majority applications of aAPC in CAR-T cell therapy are for hematological tumour. Recently, we proposed a new strategy in aAPC to treat solid tumours [62]. This involves co-infusion of CAR-T cells with aAPCs that express chemokines and costimulatory ligands. This approach enhances the antitumour effect in mice. We also found that a cytokine cocktail (IL-2, IL-9 and IL-21) can effectively amplify the anti-tumour effect. Conceptually, aAPC system is an appealing platform for taking advantage of the simple genetic modification of cells such as K562. aAPC can thus improve T cell function and overcome several challenges encountered by CAR-T in the treatment of solid tumours.

### Application of cell-based aAPC in TIL therapy

Tumour infiltrating lymphocyte (TIL) adoptive therapy are to collect T lymphocytes from the tumour site, which is expanded by in vitro culture, and then infused into patients [112]. However, the in vitro expansion of autologous TIL requires a large number of allogeneic PBMC cells as feeder cells, which is time-consuming and costly. Therefore, aAPC as an off-the-shelf “feeder cells” is also one of the effective means for rapid expansion of TIL [113, 114]. Co-expressed a membrane-bound monoclonal antibody (anti-CD3 antibody) and CD80 and CD83 on K562 cells to generate a novel aAPC [115]. This aAPCs efficiently amplified not only CD3^+^ peripheral T lymphocytes but also T lymphocytes recovered from ascites in breast and ovarian cancer patients. The aAPC platform was used to rapidly amplify tumour-infiltrating lymphocytes from primary tumour specimens, based on the Carl H June designed aAPC-K32, and further co-transfection with co-stimulatory molecule 4-1BBL and allowing it to secrete IL-2, IL-15, or IL-21 cytokines [116]. These amplified TIL are more central memory and effector memory phenotypes, and also highly express molecules such as CD27 and CD28 that are associated with the survival and persistence of T cells in vivo. Treg cells do not respond to stimulation of this aAPC, and thus this aAPC is believed to efficiently amplify functional TIL without causing immunosuppression [117].

### Application of cell-based aAPC in CTL therapy

Antigen-specific cytotoxic T lymphocytes are also one of the promising immunotherapies for cancer treatment [118]. However, existing approaches for increasing CTL can take months or longer, posing a substantial constraint in cancer treatment. Many studies have demonstrated that aAPC can rapidly amplify antigen-specific CTL. *Drosophila* cells transfected with MHC-class I molecules are able to induce autologous spleen cells to respond to tumour-specific peptides in vitro. And CTL produced by this aAPC system was able to inhibit mouse P815 mastocytoma growth [119]. Another institution used K562-CD32-4-1BBL cells coated with anti-CD3/28 antibodies as aAPC to induce long-term growth of antigen-specific CTL. They found that expressing 4-1BBL on K562 helped reduce the apoptosis of CD8^+^ T cells, increasing T cell persistence and cytolytic capacity [46]. Several studies have demonstrated aAPC generated by co-expression of HLA-A2, CD80 and CD83 on K562 cells can be used to amplify long-lasting antigen-specific CTLs. The presence of CD83 maintains low expression of the depletion molecules LAG-3, PD-1, CTLA-4 and TIM-3 on CD8^+^ T cells, which favors the enrichment of antigen-specific T cells. These enriched antigen-specific CD8^+^ T cells have potent lysis effects on a wide range of melanoma cell [20, 22]. Based on their experience in preparing aAPCs to amplify CD8^+^ T cells, this team also generated a novel aAPC to stimulate HLA-DR-restricted antigen-specific CD4^+^ T cells. K562 has been engineered to express HLA-DR as a single HLA allele in addition to invariant chain (Ii), HLA-DM, CD80 and CD83 [26]. These results suggest that K562-based aAPC may serve as a translational platform for the generation of both antigen-specific CD8^+^ CTL and CD4^+^ T cells.

### Application of cell-based aAPC in NK cell therapy

NK are innate lymphocytes that can identify and remove virus-infected and tumour cells. NK can recognize targets without HLA restriction, making them appealing candidates for universal cellular immunotherapy. K562 cells are widely used in the production of NK cells because they do not express HLA molecules and do not factor the KIRs [78, 120, 121]. Membrane-bound IL-15 and 4-1BBL co-expressed on K562 cells acted synergistically to expand peripheral blood NK cells without concomitant growth of T lymphocytes [63]. Notably, aAPC expanded CAR-NK also showed significant anti-leukemia activity [64]. The K562-aAPC, which co-express CD64, CD86, 4-1 BBL, a truncated CD19 molecule and membrane-bound IL-21, were employed to facilitate NK cell proliferation [65]. This aAPC allows the generation of large numbers of activated NK cells from PBMC of children with high-risk neuroblastoma. In vitro, these NK cells are highly cytotoxic to multidrug-sensitive and resistant neuroblastoma cell lines, either alone or in combination with mAb ch14.18, and secrete a variety of antitumour cytokines and chemokines when mediating ADCC.

Human invariant natural killer T (iNKT) cells express invariant TCR Vα24, which pairs with TCR Vβ11 and recognizes glycolipids on monotype CD1d. Thus, tumour-specific iNKT cells can attack CD1d^+^ tumour cells, such as leukemia and lymphoma, without HLA restriction [122, 123]. iNKT cells were amplified using aAPCs produced by transferring CD1d, CD80, and CD83 into K562 cells [124]. These aAPCs were used to pulse synthetic ligands, such as α-Galactosyl ceramide, in order to amplify highly polyclonal iNKT cells containing various CDR3β sequences. The iNKT cells that have been amplified secrete IFN-γ and/or IL-4 in a CD1d-dependent manner. Furthermore, their subpopulations recognize endogenous ligands presented by CD1d and are self-reactive.

## Clinical research on the cell-based aAPC

Currently, only aAPCs based on *Drosophila* and K562 can be used under GMP conditions, and their safety and tolerability have been demonstrated in a number of clinical trials (Table 2) [125, 126]. To study the distribution and toxicity of CTLs against a single melanoma epitope. *Drosophila* cells transduced with HLA-A2.1, CD80, and ICAM-1 were used for priming, followed by two rounds of immunization with mononuclear cells as antigen-presenting cells [72]. In this trial, partial responses were observed following CTLs therapy. PCR amplification used to detect *Drosophila melanogaster* cells prior to returning the cells to the patient showed that the washing procedure effectively removed aAPCs.

K562 cell-based aAPCs are more widely used in clinical trials for ACT. aAPC co-expressing CD64 and 4-1BBL on K562 were used to amplify CTL for Phase I melanoma clinical study (NCT01369875). Another clinical study on melanoma was conducted by Naoto Hirano’s team (NCT00512889), based on preclinical study data [22], they generated an aAPC-A2 clone33 co-expressing HLA class I, CD80, and CD83 for amplification of HLA Class I restricted T cells. In the previous stage, they amplified TIL cells with melanoma-associated antigen MART1 positive using aAPC-A2 clone33, and the amplified TIL cells have achieved good results in terms of in vitro proliferation and specific anti-tumour effects. At present, no response has been seen in melanoma patients treated by these two clinical treatments, and those studies have been closed. The Baylor College of Medicine team has designed a clinical-grade aAPC: transduction of CD32, CD80, CD86, CD83 and the co-stimulatory molecule 4-1BBL on K562 to amplify EBV-specific T cells for Phase I treatment of EBV-specific lymphoma (NCT01555892). This approach offers the potential to replace the use of autologous EBV-transformed B cells as APCs. This provides a simpler and more convenient method of adopting T-cell therapy for EBV, VZV, vaccinia virus, cytomegalovirus, and adenovirus-associated diseases. Cooper’s group prepared clinical grade clone # 4 for the generation of clinically relevant functional CD19-specific CAR-T cells. Clinical studies using this aAPC have undergone successful FDA review and three clinical studies have been conducted for the treatment CD19^+^ hematologic malignancies (NCT01497184, NCT01362452, NCT01653717).

Several research groups are using K562-based aAPCs to generate large numbers of NK cells for the treatment of hematological cancers. The Center for Cell and Gene Therapy at Baylor College of Medicine manufactured aAPC, K562-mbIL15-4-1BBL, which was first reported by Campana and colleagues at St. Jude Children’s Research Hospital [64, 127]. NK cells amplified by the K562-mbIL15-4-1BBL were used to treat recurrent high-risk multiple myeloma in asymptomatic but high-risk patients or in combination with bortezomib. Clinical-grade aAPC (Clone #9) expressing CD64, CD86, CD19, CD86, 4-1BBL, and membrane-bound IL-21 was used for NK cell amplification in another set of NK studies at MD Anderson. The MD Anderson team is focusing on using NK cells to treat CLL, multiple myeloma, and myeloid leukemia 5–8 days before stem cell infusion. As previously stated, the phenotype and cytotoxicity of NK cells amplified with aAPC (Clone #9) were comparable to that of aAPC expressing IL-15 rather than IL-21, but with superior cytokine secretion and proliferation [128, 129]. Unfortunately, there is no evidence that aAPC produces NK cells with superior transplant or antitumour function in vivo.

Treg cell expansion with aAPC has also been used to prevent graft-versus-host-reaction (GVHD). Blazar and colleagues used lentivirus gene transfer to create clinical-grade KT64/86, K562 cells that express CD86 and CD64 [130]. In the presence of IL-2 and rapamycin, KT64/86 cells were loaded with anti-CD3 monoclonal antibodies and used to amplify purified, polyclonal, human natural Treg cells. KT64/86 amplified natural Treg cells were shown to be effective in alleviating GVHD in a xenogeneic model. These findings support the ultimate goal of generating a library of Tregs for off-the-shelf therapies to prevent and treat GVHD and transplant rejection using KT64/86 from multiple HLA donor Treg cells. Their current study (NCT00602693) involved + 1 day after cord blood stem cell transplantation. In this study, aAPC-generated Treg cells were given to a group of patients who were compared to a previous cohort of patients who had previously received CD3/CD28 beaded dilated Treg cells under the same regimen.

Taken together, cell-based aAPC can be used as a reliable and safe material for the expansion of adoptive cells for the treatment of tumours and/or GVHD.

**Table 2:** Clinical application of cell-based aAPC in ACT

Cells	Transduced molecules	Target cell for expansion	Clinical trials phase and status	Target disease
*Drosophila* cell	HLA-A2.1, CD80 and CD54	CD8^+^ CTL	Phase I Status: completed	Tyrosinase-positive melanomas
NIH/3T3	N/A			
K562	aAPC-A2, clone 33: HLA class I, CD80, CD83	Autologous aAPC-generated MART1^+^ T cells	NCT00512889, phase I Status: completed	Melanoma (Skin)
	7F11ECCE: CD64, 4-1BBL	TIL	NCT01369875, phase II Status: terminated (premature closure. protocol did not meet its primary objective.)	Metastatic melanoma Skin cancer
	K562cs: CD32, CD80, CD83 CD86, 4-1BBL	EBV-specific T cell	NCT01555892, phase I Status: recruiting	EBV-positive lymphoma: Hodgkin’s disease, non-Hodgkin’s lymphoma, lymphoproliferative disease, lymphoma
	aAPC (clone #4): CD64, CD86, 4-1BBL, truncated CD19, mbIL-15	Autologous CD19-specific CAR-T cells	NCT00968760, phase I Status: completed NCT01497184, phase I Status: completed NCT01362452, phase I Status: completed NCT01653717, phase I Status: completed	B-lineage lymphoid malignancies After umbilical cord SCT B-lineage lymphoid malignancies After auto SCT B-lineage lymphoid malignancies After allo-SCT B-cell chronic lymphocytic leukemia
	4-1BBL, mbIL-15	Autologous NK cells	NCT01884688 Phase I Status: completed	Asymptomatic multiple myeloma
	4-1BBL, mbIL-15	Autologous NK cells plus bortezomib	NCT01313897 Phase II Status: completed	Relapsed high risk multiple myeloma
	aAPC (clone #9): CD64, CD86, 4-1BBL, truncated CD19, mbIL-21	Autologous NK cells	NCT01729091 Phase I Status: unknown	B-cell chronic lymphocytic leukemia undergoing umbilical cord SCT, multiple myeloma undergoing umbilical cord SCT, and myeloid leukemia undergoing SCT
	KT64/86: CD64, CD86	Natural Treg from umbilical cord cells	NCT00602693 Phase I Status: completed	Advanced hematologic malignancies with umbilical cord SCT

*CTL* cytolytic T lymphocytes cells, *TIL* tumour infiltrating lymphocytes, *CAR* chimeric antigen receptor, *NK* natural killer, *SCT* stem cell transplantation

## Conclusions

Cell-based aAPC is an appealing material for in vitro cell expansion in ACT. However, the application of cellular reagents has major limitations, including the possibility of contamination, immunogenicity, and tumourigenicity. To address contamination during the preparation of clinical-grade aAPCs, a standard preparation code and strict release criteria can be established. The risk of contamination can include culturing cells in culture isolator. Rigorous testing for viruses, mycoplasmas and bacteria before using aAPC-expanded T cells or NK cells is also essential [32]. Using xenogeneic NIH/3T3 cells or *Drosophila* *melanogaster* cells as aAPCs has the benefit of being genetically edited to express only one engineered HLA molecule, thereby avoiding alloreactivity. Studies have shown that NIH/3T3 cells rarely activate human T cells, implying that significant alloreactivity is less likely to occur when using xenogeneic aAPC [42, 73]. The immunogenicity of xenogeneic cells cannot be avoided due to the presence of xenogeneic antigens. However, recent clinical studies have demonstrated that washing procedures can remove the insect antigens released by *Drosophila* *melanogaster*, which do not cause any issues [72]. Before using cell-based aAPCs, their proliferative potential was lost by irradiation, thus eliminating the risk of tumourigenicity [39]. There was no evidence that aAPC caused tumour formation or other adverse effects when T cells expanded with mouse fibroblast aAPC were reinfused into mice. Studies have shown that after 5 days of co-culture with T cells, neither flow cytometry nor RT-PCR could detect the remaining K562 cells. This meant that aAPC could be completely eliminated from the culture [115]. The safety of irradiated cells has also been demonstrated in a number of tumour vaccine clinical trials [126]. However, the clinical study of aAPC for ACT is still in the early stages, and more clinical data is required to support its safety.

Acellular systems are currently being used as an alternative to cellular aAPC. Various acellular aAPC systems, including beads, liposomes, and exosomes, have been developed [81]. However, contact and signaling between acellular aAPC and immune cells rely entirely on artificial modification. Cellular aAPCs have a primary advantage over acellular aAPCs in their ability to effectively simulate physiological interactions between T cells and APCs. These interactions are essential for aAPC signaling of T cells, particularly in terms of allowing the migration of molecules required to activate T cells [12]. Naturally expressed adhesion molecules on K562 cells, for example, aid in the formation of immune synapses between T cells and aAPC, which is absolutely essential for aAPC signaling of T cells. The signaling of acellular aAPC to T cells is also affected by the characteristics of the T cells themselves [81]. The most commonly used aAPC system is antibody-coated beads, which are simple but may have limited function due to their mechanical rigidity and limited ability to display different ligands. Liposome aAPC function is also influenced by its size and stability [11, 81].

The primary goal of aAPC development is to create an alternative system for providing effective activation and proliferation signals for in vitro adoptive cell culture. This is achieved by avoiding the difficult-to-isolate and preparing professional APCs. The properties of amplified T cells are not limited to cell number, phenotype, and in vitro lysis activity. The signals sent by aAPCs must be strong enough to stimulate proliferation but not strong enough to induce AICDs for terminal differentiation or high-affinity T cells. Developing appropriate aAPC for the production of adoptive cells with optimal function is a future research trend and challenge. Currently, cellular aAPC is primarily created through gene modification to express three signals required for T cell activation: the TCR-pMHC complex, co-stimulatory signaling, and cytokines [131]. In addition to signals that activate T cells, there are also inhibitory signals, such as apoptosis-inducing molecules, that deplete against antigen-specific T cells. One advantage of aAPCs is their ability to be genetically modified to express various co-stimulatory or inhibitory molecules, which is useful for analyzing different T cell responses and improving adoptive T cell function. Additionally, Schoenberger, S.P. et al. conducted a study on the requirements for antigen density and co-stimulatory molecules during direct CTL initiation with aAPC. Properly engineered aAPCs can efficiently expand adoptive cells without the risk of cross-reactions [40]. In summary, aAPC is not only an efficient method for expanding adoptive cells in vitro, but it also provides a good platform for elucidating the biological requirements of T cells with high therapeutic potency.

## Data Availability

Not applicable.
